# Supplementary material for: Multi‐dataset identification of innovative feature genes and molecular mechanisms in keratoconus
Source: J Cell Mol Med. 2024 Sep 19;28(18):e70079. doi: 10.1111/jcmm.70079 (PMC11412914; doi:10.1111/jcmm.70079)
Supplement: Supplementary file 2 — Material S2. [file JCMM-28-e70079-s002.zip › 7.GSEA analysis/5.RNF217-AS1/KEGG_TGF_BETA_SIGNALING_PATHWAY.html]

Details for gene set KEGG\_TGF\_BETA\_SIGNALING\_PATHWAY[GSEA]

|  || Dataset | RNF217-AS1.group.cls#h\_versus\_l |
| Phenotype | group.cls#h\_versus\_l |
| Upregulated in class | l |
| GeneSet | KEGG\_TGF\_BETA\_SIGNALING\_PATHWAY |
| Enrichment Score (ES) | -0.3888947 |
| Normalized Enrichment Score (NES) | -1.2932394 |
| Nominal p-value | 0.15068494 |
| FDR q-value | 0.4047585 |
| FWER p-Value | 0.958 |
Table: GSEA Results Summary

  

Fig 1: Enrichment plot: KEGG\_TGF\_BETA\_SIGNALING\_PATHWAY      
 Profile of the Running ES Score & Positions of GeneSet Members on the Rank Ordered List

  

| SYMBOL | TITLE | RANK IN GENE LIST | RANK METRIC SCORE | RUNNING ES | CORE ENRICHMENT || 1 | CDKN2B | na | 2482 | 0.317 | -0.0406 | No |
| 2 | GDF7 | na | 2827 | 0.299 | -0.0213 | No |
| 3 | BMPR1B | na | 4415 | 0.237 | -0.0439 | No |
| 4 | THBS1 | na | 4580 | 0.231 | -0.0261 | No |
| 5 | BMP8B | na | 5147 | 0.212 | -0.0217 | No |
| 6 | AMHR2 | na | 5736 | 0.192 | -0.0200 | No |
| 7 | ACVR2B | na | 6291 | 0.174 | -0.0189 | No |
| 8 | INHBC | na | 6720 | 0.161 | -0.0155 | No |
| 9 | NOG | na | 7115 | 0.150 | -0.0122 | No |
| 10 | PPP2R1B | na | 9136 | 0.102 | -0.0605 | No |
| 11 | TGFB1 | na | 9166 | 0.102 | -0.0514 | No |
| 12 | ZFYVE9 | na | 9614 | 0.093 | -0.0552 | No |
| 13 | ROCK1 | na | 10313 | 0.081 | -0.0674 | No |
| 14 | SMAD5 | na | 10360 | 0.080 | -0.0608 | No |
| 15 | BMP8A | na | 10795 | 0.073 | -0.0662 | No |
| 16 | ACVRL1 | na | 11269 | 0.066 | -0.0734 | No |
| 17 | SMURF1 | na | 12661 | 0.047 | -0.1090 | No |
| 18 | MAPK1 | na | 13362 | 0.038 | -0.1255 | No |
| 19 | TNF | na | 13583 | 0.035 | -0.1284 | No |
| 20 | THBS3 | na | 13787 | 0.033 | -0.1310 | No |
| 21 | MAPK3 | na | 14127 | 0.029 | -0.1380 | No |
| 22 | SP1 | na | 14246 | 0.028 | -0.1386 | No |
| 23 | CREBBP | na | 14557 | 0.024 | -0.1452 | No |
| 24 | SMAD3 | na | 14794 | 0.021 | -0.1500 | No |
| 25 | CHRD | na | 14901 | 0.020 | -0.1511 | No |
| 26 | RHOA | na | 14935 | 0.020 | -0.1501 | No |
| 27 | PPP2CB | na | 15463 | 0.014 | -0.1639 | No |
| 28 | SMAD2 | na | 16024 | 0.008 | -0.1792 | No |
| 29 | GDF6 | na | 16136 | 0.007 | -0.1817 | No |
| 30 | RPS6KB2 | na | 16186 | 0.007 | -0.1825 | No |
| 31 | RBX1 | na | 16317 | 0.006 | -0.1857 | No |
| 32 | SKP1 | na | 16603 | 0.003 | -0.1936 | No |
| 33 | ACVR1C | na | 16626 | 0.003 | -0.1940 | No |
| 34 | SMAD4 | na | 16849 | 0.000 | -0.2004 | No |
| 35 | BMPR2 | na | 16870 | -0.000 | -0.2010 | No |
| 36 | PPP2R1A | na | 17564 | -0.007 | -0.2203 | No |
| 37 | RPS6KB1 | na | 17704 | -0.008 | -0.2235 | No |
| 38 | RBL2 | na | 18022 | -0.011 | -0.2316 | No |
| 39 | ID1 | na | 18373 | -0.014 | -0.2403 | No |
| 40 | TGFBR2 | na | 18634 | -0.017 | -0.2462 | No |
| 41 | ROCK2 | na | 18737 | -0.018 | -0.2474 | No |
| 42 | BMP5 | na | 18797 | -0.018 | -0.2473 | No |
| 43 | TFDP1 | na | 18838 | -0.019 | -0.2466 | No |
| 44 | PPP2CA | na | 19715 | -0.027 | -0.2692 | No |
| 45 | ACVR2A | na | 20110 | -0.031 | -0.2776 | No |
| 46 | BMPR1A | na | 20776 | -0.038 | -0.2931 | No |
| 47 | LEFTY1 | na | 20861 | -0.038 | -0.2918 | No |
| 48 | LTBP1 | na | 21232 | -0.042 | -0.2983 | No |
| 49 | INHBA | na | 21567 | -0.046 | -0.3035 | No |
| 50 | BMP7 | na | 21966 | -0.050 | -0.3100 | No |
| 51 | CUL1 | na | 21972 | -0.050 | -0.3052 | No |
| 52 | SMAD7 | na | 23250 | -0.065 | -0.3358 | No |
| 53 | EP300 | na | 23570 | -0.069 | -0.3382 | No |
| 54 | ZFYVE16 | na | 24076 | -0.076 | -0.3453 | No |
| 55 | TGFB2 | na | 24475 | -0.082 | -0.3487 | No |
| 56 | NODAL | na | 24519 | -0.083 | -0.3419 | No |
| 57 | SMAD1 | na | 24724 | -0.086 | -0.3393 | No |
| 58 | RBL1 | na | 24761 | -0.087 | -0.3319 | No |
| 59 | BMP2 | na | 25185 | -0.093 | -0.3350 | No |
| 60 | ACVR1 | na | 26536 | -0.117 | -0.3625 | No |
| 61 | THBS2 | na | 26660 | -0.119 | -0.3544 | No |
| 62 | TGFBR1 | na | 27639 | -0.140 | -0.3690 | No |
| 63 | E2F4 | na | 28238 | -0.155 | -0.3711 | No |
| 64 | GDF5 | na | 28855 | -0.172 | -0.3721 | Yes |
| 65 | ID3 | na | 29042 | -0.177 | -0.3601 | Yes |
| 66 | INHBE | na | 29119 | -0.180 | -0.3447 | Yes |
| 67 | SMURF2 | na | 29259 | -0.184 | -0.3308 | Yes |
| 68 | AMH | na | 29321 | -0.185 | -0.3144 | Yes |
| 69 | BMP6 | na | 30045 | -0.208 | -0.3150 | Yes |
| 70 | BMP4 | na | 30086 | -0.209 | -0.2957 | Yes |
| 71 | FST | na | 30586 | -0.226 | -0.2879 | Yes |
| 72 | SMAD9 | na | 30728 | -0.231 | -0.2694 | Yes |
| 73 | MYC | na | 30784 | -0.234 | -0.2482 | Yes |
| 74 | ID4 | na | 30961 | -0.241 | -0.2297 | Yes |
| 75 | LEFTY2 | na | 31836 | -0.282 | -0.2274 | Yes |
| 76 | E2F5 | na | 31895 | -0.284 | -0.2013 | Yes |
| 77 | TGFB3 | na | 32072 | -0.296 | -0.1774 | Yes |
| 78 | SMAD6 | na | 32100 | -0.297 | -0.1492 | Yes |
| 79 | DCN | na | 32159 | -0.301 | -0.1214 | Yes |
| 80 | THBS4 | na | 32170 | -0.301 | -0.0923 | Yes |
| 81 | INHBB | na | 32228 | -0.305 | -0.0641 | Yes |
| 82 | ID2 | na | 32256 | -0.307 | -0.0349 | Yes |
| 83 | COMP | na | 32268 | -0.307 | -0.0052 | Yes |
| 84 | PITX2 | na | 33321 | -0.387 | 0.0023 | Yes |
| 85 | IFNG | na | 33359 | -0.391 | 0.0395 | Yes |
Table: GSEA details [plain text format]

  

Fig 2: KEGG\_TGF\_BETA\_SIGNALING\_PATHWAY      
 Blue-Pink O' Gram in the Space of the Analyzed GeneSet

  

Fig 3: KEGG\_TGF\_BETA\_SIGNALING\_PATHWAY: Random ES distribution      
 Gene set null distribution of ES for **KEGG\_TGF\_BETA\_SIGNALING\_PATHWAY**
